# The Bidirectional Relationship Between Iron Deficiency Anemia and Chronic Headache Disorders: A Systematic Review and Meta-Analysis

**DOI:** 10.1155/anem/5695022

**Published:** 2025-02-07

**Authors:** Shivam Rohilla, Ganesh Bushi, Mahalaqua Nazli Khatib, Suhas Ballal, Pooja Bansal, Balvir S. Tomar, Ayash Ashraf, Ravi Kumar M., Aashna Sinha, Pramod Rawat, Abhay M. Gaidhane, Sanjit Sah, Hashem Abu Serhan, Muhammed Shabil

**Affiliations:** ^1^Department of Pharmacy Practice, National Institute of Pharmaceutical Education and Research, Guwahati, India; ^2^Center for Global Health Research, Saveetha Medical College and Hospital, Saveetha Institute of Medical and Technical Sciences, Saveetha University, Chennai, India; ^3^School of Pharmaceutical Sciences, Lovely Professional University, Phagwara 144411, India; ^4^Division of Evidence Synthesis, Global Consortium of Public Health and Research, Datta Meghe Institute of Higher Education, Wardha, India; ^5^Department of Chemistry and Biochemistry, School of Sciences, JAIN (Deemed to be University), Bangalore, Karnataka, India; ^6^Department of Allied Healthcare and Sciences, Vivekananda Global University, Jaipur Rajasthan-303012, India; ^7^Institute of Pediatric Gastroenterology and Hepatology, NIMS University, Jaipur, India; ^8^Department of Pharmacy, Chandigarh Pharmacy College, Chandigarh Group of College, Jhanjeri, Mohali 140307, Punjab, India; ^9^Department of Chemistry, Raghu Engineering College, Visakhapatnam Andhra Pradesh-531162, India; ^10^School of Applied and Life Sciences, Division of Research and Innovation, Uttaranchal University, Dehradun, India; ^11^Department of Biotechnology, Graphic Era (Deemed to be University), Clement Town, Dehradun 248002, India; ^12^Department of Allied Sciences, Graphic Era Hill University, Clement Town, Dehradun 248002, India; ^13^Jawaharlal Nehru Medical College and Global Health Academy, School of Epidemiology and Public Health, Datta Meghe Institute of Higher Education, Wardha, India; ^14^Department of Paediatrics, Dr. D. Y. Patil Medical College, Hospital and Research Centre, Dr. D. Y. Patil Vidyapeeth, Pune 411018, Maharashtra, India; ^15^Department of Public Health Dentistry, Dr. D.Y. Patil Dental College and Hospital, Dr. D.Y. Patil Vidyapeeth, Pune 411018, Maharashtra, India; ^16^Department of Medicine, SR Sanjeevani Hospital, Kalyanpur, Siraha 56517, Nepal; ^17^Department of Ophthalmology, Hamad Medical Corporation, Doha, Qatar; ^18^Center for Research and Development, University Center for Research and Development, Chandigarh University, Mohali, Punjab, India; ^19^Medical Laboratories Techniques Department, AL-Mustaqbal University 51001, Hillah, Babil, Iraq

**Keywords:** bidirectional relationship, chronic headache, IDA, meta-analysis, migraine, systematic review

## Abstract

**Background and Objective:** IDA and chronic headache disorders such as migraines and tension-type headaches are common conditions that significantly affect quality of life. Emerging evidence suggests a bidirectional relationship between these two conditions. This systematic review and meta-analysis aimed to explore and quantify the association between iron deficiency anemia (IDA) and chronic headache disorders, with a focus on understanding the bidirectional nature of this relationship.

**Methods:** A comprehensive literature search was conducted across PubMed, Embase, and Web of Science to identify relevant studies published up until August 10, 2024. Observational studies examining the prevalence, incidence, or association between IDA and chronic headache disorders were included. Data were extracted and assessed for quality using the Newcastle–Ottawa Scale. Meta-analyses were performed using a random-effects model to calculate pooled prevalence rates and risk ratios (RRs), with heterogeneity assessed via the *I*^2^ statistic and meta-regression. A sensitivity analysis was conducted using the leave-one-out approach, and publication bias was evaluated through a funnel plot.

**Results:** The meta-analysis included 13 studies: five studies examined chronic headaches among patients with IDA, and eight studies examined IDA among patients with chronic headaches. The pooled prevalence of chronic headaches among patients with IDA was 38% (95% CI: 15%–69%). In addition, 20% (95% CI: 10%–35%) of patients with chronic headaches were found to have IDA. Anemic patients were found to have a 76% higher risk of developing chronic headaches compared to nonanemic individuals (RR: 1.76; 95% CI: 1.22–2.52). Significant heterogeneity was observed across the studies.

**Conclusion:** This meta-analysis demonstrates a significant association between IDA and chronic headache disorders, with a pooled prevalence of 38% for chronic headaches in IDA patients and 20% for IDA in chronic headache patients. IDA was associated with a 76% higher risk of chronic headaches. Routine screening for IDA in high-risk populations may improve headache outcomes, but further longitudinal studies are needed to establish causality and refine management strategies.

## 1. Introduction

Chronic headache disorders, including migraines and tension-type headaches, are among the most common neurological conditions worldwide, significantly affecting individuals' quality of life and imposing substantial socioeconomic costs [1, 2]. These disorders are characterized by frequent and often debilitating episodes of pain that disrupt daily activities and reduce productivity. While the multifactorial origins of chronic headache disorders are well-documented, recent studies have begun to explore a potential bidirectional relationship with iron deficiency anemia (IDA), a prevalent hematological condition marked by reduced hemoglobin levels and compromised oxygen transport to bodily tissues [3, 4]. The established link between IDA and neurological symptoms, including headaches, is crucial for understanding chronic headache disorders. IDA directly impacts cerebral oxygenation and neurotransmitter synthesis, both pivotal in headache pathophysiology. In contrast, other anemias, such as megaloblastic and hemolytic anemias, involve distinct mechanisms such as vitamin deficiencies and rapid red blood cell destruction that can lead to neurological issues [5, 6] Therefore, our study primarily focuses on IDA.

IDA impacts multiple organ systems, notably the central nervous system. Symptoms such as fatigue, cognitive impairment, and headaches suggest that IDA could worsen or even initiate chronic headache disorders [7]. Conversely, the management of chronic headaches often involves medications, such as nonsteroidal anti-inflammatory drugs (NSAIDs), which are known to cause gastrointestinal bleeding and potentially lead to IDA. Moreover, the persistent inflammation associated with chronic headaches could disrupt iron metabolism, exacerbating anemia [8].

The suggested bidirectional relationship between IDA and chronic headaches mediated through mechanisms such as cerebral hypoxia, altered neurotransmitter activity, and increased cerebral blood flow highlights the complexity of these conditions. Addressing one may necessitate managing the other to effectively break the cycle of mutual exacerbation. However, despite its clinical significance, research exploring this interplay remains sparse. Existing studies typically focus on IDA or chronic headaches in isolation, rarely examining their reciprocal dynamics.

Current clinical guidelines for both IDA and chronic headaches lack comprehensive strategies that address their interconnection. This oversight can lead to suboptimal patient outcomes, as treatments targeting only one condition may inadvertently aggravate the other [9–13]. This systematic review and meta-analysis seek to fill these critical gaps by synthesizing existing evidence to clarify the bidirectional prevalence and association between IDA and chronic headache disorders. By integrating these findings, our study aims to illuminate the underlying mechanisms and interactions, fostering a holistic approach to diagnosis, treatment, and prevention that could enhance patient care and improve quality of life. This endeavor not only aims to validate the hypothesized links but also to establish a foundation for future guidelines that can more effectively manage these intertwined conditions.

## 2. Methods

This systematic review and meta-analysis were conducted in accordance with the Preferred Reporting Items for Systematic Reviews and Meta-Analyses (PRISMA) 2020 guidelines (Table S1) [14]. The protocol for this review was registered in the PROSPERO database.

### 2.1. Eligibility Criteria

Studies were eligible for inclusion if they met the following criteria [1]: involved human participants of any age or sex [2]; examined the prevalence, incidence, or association between anemia (specifically IDA) and chronic headache disorders (including migraines and tension-type headaches) as defined by the ICD-10 guidelines, where tension-type headaches and migraines are categorized under chronic headaches [3]; employed observational study designs, including cross-sectional, case-control, and cohort studies [4]; were published in peer-reviewed journals; and [5] were available in English. Exclusion criteria included studies with insufficient data to extract relevant outcomes, reviews, case reports, editorials, and studies focusing exclusively on secondary headache disorders or anemia caused by conditions other than iron deficiency (Table S2).

### 2.2. Database Search

A comprehensive literature search was conducted in multiple electronic databases, including PubMed, Embase, and Web of Science, to identify relevant studies published up until August 10, 2024. The search strategy combined keywords and Medical Subject Headings (MeSH) terms related to IDA (e.g., “anemia” and “IDA”) and chronic headache disorders (e.g., “migraine,” “tension-type headache,” and “chronic headache”). No date restrictions were applied. The full search strategies for each database are detailed in Supporting Table S3.

### 2.3. Screening and Data Extraction

The initial screening of titles and abstracts, followed by full-text reviews, was performed independently by two reviewers using Nested Knowledge software. Discrepancies between reviewers were resolved through discussion or by consulting a third reviewer. Data extraction was also conducted using the Nested Knowledge software, ensuring a standardized approach. Extracted data included study characteristics (e.g., author, year, country, study design, and sample size), participant demographics (e.g., age and sex), definitions and measurements of IDA and headache disorders, outcomes related to the prevalence and association of IDA and chronic headache disorders, and relevant confounding factors.

### 2.4. Quality Evaluation

Study quality was appraised using the Newcastle–Ottawa Scale (NOS) for cohort studies, assessing participant selection, group comparability, and exposure/outcome assessment (Table S4). Each study received a score from 0 to 9; scores of 7 or higher indicated high quality, scores of 5-6 suggested moderate quality, and scores below 5 denoted low quality. This evaluation was independently performed by two reviewers, with discrepancies resolved by consensus.

### 2.5. Evidence Synthesis

Statistical analyses were performed using R Version 4.4, employing the “metafor” package for meta-analysis. A random-effects model was used to calculate pooled estimates of prevalence and risk ratios (RRs) with 95% confidence intervals (CIs), considering the anticipated heterogeneity among studies [15]. Heterogeneity was assessed using the Baujat plot, meta-regression, and the *I*^2^ statistic, with values greater than 50% indicating substantial heterogeneity. Leave-one-out sensitivity analyses were conducted to explore the robustness of the findings, and publication bias was assessed using a funnel plot [16].

## 3. Results

### 3.1. Literature Search

**Figure 1:**
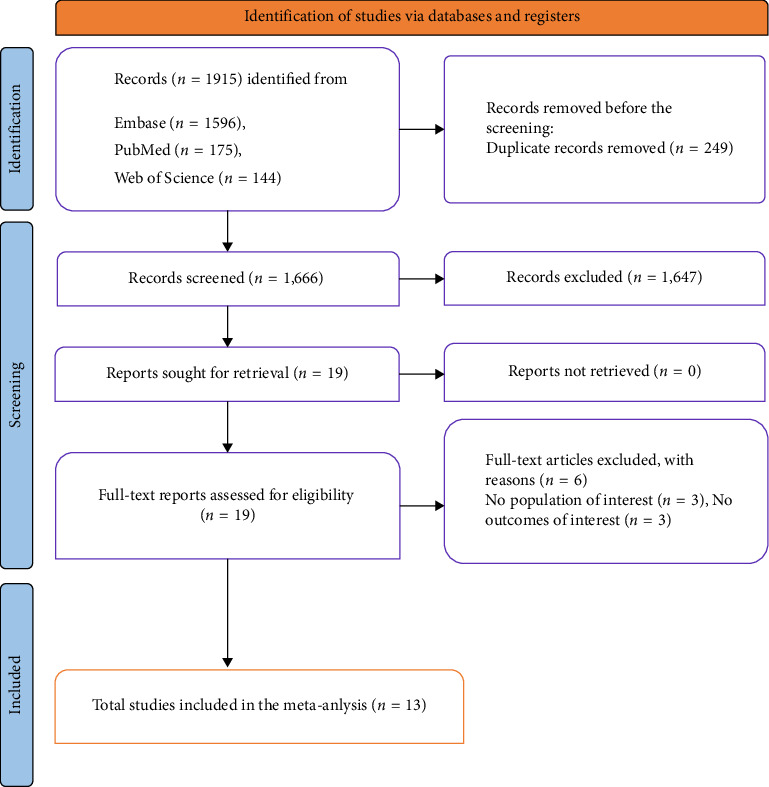
PRISMA flowchart.

Through literature searches, 1915 records were identified in Embase, PubMed, and Web of Science (see Figure 1 PRISMA flow diagram). After removing 249 duplicates, 1666 records were screened, leading to the exclusion of 1647 based on titles and abstracts. Full texts of 19 reports were assessed for eligibility, with five studies excluded due to either not involving the population of interest (*n* = 3) or not reporting relevant outcomes (*n* = 3). Ultimately, 13 studies met the inclusion criteria and were included in the final analysis: 8 studies examined IDA in chronic headache disorders, and 5 studies examined chronic headache disorders in IDA.

### 3.2. Study Characteristics

Five studies [9–11, 17, 18], with sample sizes ranging from 142 to 2385 participants, assessed the association between various types of IDA and chronic headaches or migraines (Table 1). These studies were conducted across Turkey, Iran, Norway, and Nigeria and employed diverse designs, including cross-sectional, retrospective, and quasiexperimental approaches. Participant ages ranged from adults with mean ages of 27.05–43.3 years, and male representation varied from 0% to 43%. The types of anemia studied were defined by low hemoglobin levels. In addition, eight studies [3, 12, 13, 19–23] (Table 2) examined the prevalence of anemia among patients with chronic headaches across India, the United States, Turkey, Israel, Germany, and Iran. Sample sizes varied from 100 to 10,918 participants. These studies focused primarily on IDA and its association with various chronic headache types, including menstrual migraine, tension-type headache, and chronic daily headache (CDH), with participant ages ranging from children (mean age: 11.6 years) to adults (mean ages: 36.28–51.1 years).

### 3.3. Meta-Analysis

#### 3.3.1. Prevalence of Chronic Headache Among Patients With IDA

**Figure 2:**
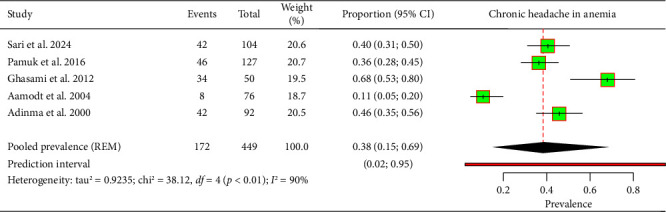
Forest plot depicting chronic headaches among patients with IDA.

The meta-analysis, which included data from five studies encompassing 449 patients with IDA, identified a pooled prevalence of chronic headache in this population of 38% (95% CI: 15%–69%) (Figure 2). The prediction interval was broad, ranging from 2% to 95%, indicating substantial variability across different populations and settings. Significant heterogeneity was observed among the studies (*I*^2^ = 90%).

#### 3.3.2. Prevalence of IDA Among Patients With Chronic Headache

**Figure 3:**
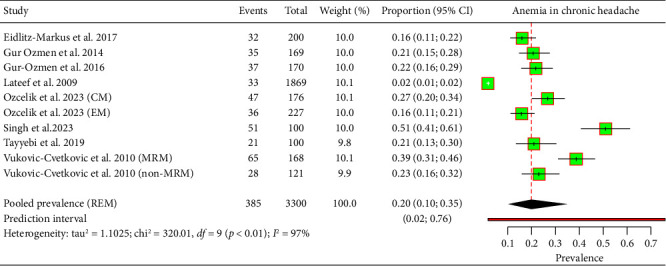
Forest plot illustrating IDA among patients with chronic headaches.

The meta-analysis, which included eight studies and a total of 3300 patients, assessed the prevalence of IDA among those with chronic headaches, finding a pooled prevalence of 20% (95% CI: 10%–35%) (Figure 3). The wide prediction interval, ranging from 2% to 76%, and a high heterogeneity index (*I*^2^ = 97%) reflect substantial variability across different studies and populations.

#### 3.3.3. Association Between IDA and Chronic Headache

**Figure 4:**
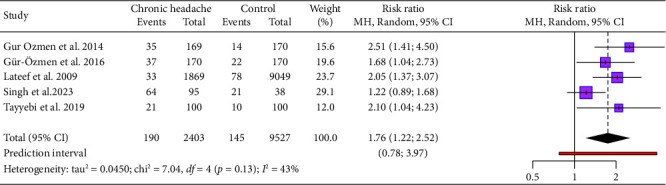
Forest plot depicting the association between chronic headache and IDA.

The meta-analysis pooled data from five studies, comprising a total of 2403 patients with chronic headaches and 9527 control participants (without headaches), to evaluate the association between IDA and chronic headaches (Figure 4). The pooled RR for IDA in chronic headache patients compared to nonheadache controls is 1.76 (95% CI: 1.22–2.52), indicating that chronic headache patients have a 76% higher likelihood of having IDA. The prediction interval ranges from 0.78 to 3.97, reflecting variability across populations. While heterogeneity is moderate (*I*^2^ = 43%, *p*=0.13), most studies report a statistically significant association, emphasizing the higher prevalence of IDA in chronic headache patients compared to controls without headaches.

### 3.4. Meta-Regression

The meta-regression analysis based on sample size for chronic headache in patients with IDA did not show a significant impact on heterogeneity (*p* value = 0.6715), indicating that variations in study size do not account for the observed differences (Figure S1). However, in the analysis of IDA in patients with chronic headaches, the *p* value of < 0.001 suggests that larger sample sizes significantly influenced the results (Figure S2).

### 3.5. Sensitivity Analysis

The leave-one-out sensitivity analyses indicate that the pooled prevalence estimates are robust and not disproportionately influenced by any single study, despite high heterogeneity. For chronic headaches in patients with IDA, the pooled prevalence remained stable at 38% (95% CI: 15%–69%), with heterogeneity (*I*^2^) ranging from 79% to 92%. The prevalence varied slightly, from 31% (when omitting [10]) to 46% (when omitting [17]) (Figure S3). For IDA in patients with chronic headaches, the pooled prevalence was consistent at 19% (95% CI: 10.4%–34.7%), with heterogeneity (*I*^2^) consistently high at 97%, reducing to 89% only when Lateef et al.'s [13] was omitted. The prevalence ranged from 17.5% (excluding [3]) to 25% (excluding [13]) (Figure S4). These findings underscore the stability of the estimates despite persistent heterogeneity. Furthermore, Baujat plots did not highlight any particular study as a major source of heterogeneity (Figures S5 and S6).

### 3.6. Publication Bias

The analyses for “chronic headache in IDA” (Figure S7) and “IDA in chronic headache” (Figure S8) demonstrate that the symmetry observed in both the funnel plots suggests that the meta-analysis results are robust and largely unaffected by any single study or potential publication bias. This provides confidence in the stability and reliability of the reported associations. Both funnel plots exhibit a generally symmetric distribution around the effect size, indicating minimal publication bias.

## 4. Discussion

This systematic review and meta-analysis sought to illuminate the bidirectional relationship between IDA and chronic headaches. The study's findings reveal a substantial association between these conditions, emphasizing the need for integrated clinical management strategies. The analysis identified a significant prevalence of chronic headaches among patients with IDA, with a pooled prevalence of 38% (95% CI: 15%–69%). In addition, 20% (95% CI: 10%–35%) of patients with chronic headache disorders were found to suffer from IDA. Furthermore, the analysis demonstrated that patients with IDA are at a 76% higher risk of developing chronic headaches compared to nonanemic individuals, as indicated by a pooled RR of 1.76 (95% CI: 1.22–2.52). These findings strongly support the existence of a bidirectional relationship between IDA and chronic headache disorders, suggesting that the presence of one condition may exacerbate or contribute to the development of the other. This study significantly advances the understanding of these conditions by highlighting their complex interplay, which has often been overlooked in clinical practice. By synthesizing data from multiple studies, this meta-analysis underscores the importance of considering IDA in the clinical management of patients with chronic headaches. It suggests that routine screening for IDA in these patients could lead to more comprehensive treatment strategies, potentially improving patient outcomes.

The significant association between IDA and chronic headache disorders found in this study is consistent with previous research that has suggested a link between these conditions. Earlier studies have demonstrated that IDA can lead to cerebral hypoxia, a well-known trigger for migraines and other types of headaches [11]. The observation that chronic headache patients exhibit a high prevalence of IDA aligns with reports indicating that the chronic use of NSAIDs, commonly used in headache management, can cause gastrointestinal bleeding, subsequently leading to IDA [24]. This bidirectional relationship, therefore, not only confirms the findings of earlier research but also underscores the need for integrated treatment approaches that address both conditions simultaneously. The findings suggest that the relationship between IDA and chronic headache disorders is more significant than previously understood, particularly in the context of IDA. Several studies have highlighted the increased risk of migraine among patients with IDA, underscoring the importance of addressing iron deficiency in the management of chronic headache disorders. For instance, a nationwide cohort study by Lee et al. demonstrated that the incidence of migraine in patients with IDA was significantly higher than in those without IDA. The study reported a cumulative incidence of 5.82 per 1000 person-years in the IDA group, compared to 3.99 per 1000 person-years in the non-IDA group, with an adjusted hazard ratio (aHR) of 1.68 (95% CI = 1.51−1.87, *p* < 0.001) after adjusting for confounding factors. These findings suggest that IDA is a significant risk factor for migraine, particularly among women and individuals under 50 years of age [25].

Moreover, the role of iron deficiency without anemia (IDWA) in headache prevalence is increasingly recognized as a crucial factor in the development of chronic headaches. A pilot study by Kita and Yamashiro explored this connection and found that headaches were more prevalent in patients with IDWA than in those with IDA [26]. The study revealed that five out of seven patients in the IDWA group presented with headaches resembling migraines or tension-type headaches, which were effectively treated with oral iron supplementation. These findings indicate that IDWA, despite normal hemoglobin levels, may still cause significant symptoms, including chronic headaches, and should therefore be considered in the diagnostic evaluation of patients with unexplained headaches, particularly women of reproductive age. The implications of these findings are significant, as they suggest that IDWA, although often not clinically apparent due to normal hemoglobin levels, can still result in substantial symptoms, including chronic headaches. This indicates the necessity for a broader diagnostic approach that includes serum ferritin measurement in patients presenting with unexplained chronic headaches, particularly in those populations most at risk, such as women of reproductive age.

The pathophysiological mechanisms underlying the association between iron deficiency and headache disorders are complex and multifactorial. They involve significant alterations in neurotransmitter function, particularly within the dopaminergic system, which plays a crucial role in migraine pathogenesis. A study by Ozmen and Ozcan [20] provided insights into this relationship through their study on menstrual-related migraine (MRM). They found a significant association between IDA and MRM, suggesting that interactions between estrogen and dopamine, both critical in iron metabolism and migraine pathogenesis, might explain the link. Iron deficiency has been shown to disrupt dopaminergic function, leading to decreased dopamine transporter density and activity, as well as a reduction in dopamine receptor expression in the striatum. This disruption may contribute to migraine development, as dopamine plays a key role in modulating pain and other migraine-related symptoms such as nausea and yawning. Furthermore, hormonal factors, particularly fluctuations in estrogen levels during the menstrual cycle, may exacerbate iron deficiency and trigger migraines. Estrogen is known to regulate iron metabolism, and its cyclical variations could contribute to the development of migraines in women with IDA. This interaction between hormonal fluctuations and iron deficiency underscores the need for a gender-specific approach to managing migraines in women, particularly those of reproductive age [12].

The clinical implications of these findings are substantial. They suggest that routine screening for iron deficiency, including IDWA, should be considered in patients with chronic headaches, especially in high-risk groups such as women of reproductive age and adolescents. These subgroups are known to experience higher rates of IDA due to menstrual blood loss and rapid growth phases, respectively, and they also report a higher prevalence of migraines and tension-type headaches. Early identification and treatment of iron deficiency could prevent the development of chronic headaches and improve patient outcomes. The findings from Keiichiro and Yamashiro [26] support this approach, suggesting that serum ferritin measurement should be included in the diagnostic workup for young women presenting with headaches. Such screening could lead to the early identification and treatment of IDWA and IDA, potentially preventing the development of chronic headaches. Moreover, iron supplementation could be a valuable therapeutic strategy for managing headaches in patients with IDA or IDWA, as evidenced by the reduction in migraine risk observed in IDA patients receiving iron supplementation in the study by Lee et al. (2020). However, the appropriate dosage and duration of iron supplementation for headache prevention remain areas for further research. In addition, the study also revealed some results that diverge from previous findings. For instance, while some studies have suggested that IDA may not significantly impact the severity of headaches, this meta-analysis found a higher prevalence of chronic headaches among anemic patients, suggesting a stronger link than previously thought. This discrepancy could be due to differences in study populations, methodologies, or the criteria used to define IDA and chronic headaches. The high heterogeneity observed in the meta-analyses further indicates that the relationship between these conditions may vary based on individual patient factors, such as age, sex, and comorbid conditions, which were not uniformly accounted for in all studies.

Despite the robust findings, this study has limitations that could influence the results. The pooled results are derived from combining retrospective, cross-sectional, and quasiexperimental study designs. The observational nature of the included studies limits the ability to draw causal inferences about the relationship between IDA and chronic headaches. While the association is clear, the directionality remains uncertain. In addition, significant heterogeneity was observed across the studies, likely due to differences in study populations, diagnostic criteria, and methodologies. A subgroup analysis between tension-type headaches and migraines was not possible due to the limited number of studies. Although a random-effects model was used to account for this variability, the findings should be interpreted with caution. Moreover, the potential for publication bias cannot be entirely ruled out, despite the minimal asymmetry observed in the funnel plot. Finally, the exclusion of non-English studies may have limited the comprehensiveness of the review, as relevant studies published in other languages were not included.

Future research should aim to address these limitations by conducting large-scale, prospective studies to better establish the causal relationship between IDA and chronic headache disorders. Longitudinal studies could help determine whether treating IDA in patients with chronic headaches reduces the frequency or severity of headache symptoms, providing more definitive evidence of a causal link. In addition, future studies should explore the impact of different types of anemia, beyond IDA, on chronic headaches to ascertain whether the observed associations are specific to IDA or extend to other forms of anemia. Separate studies focusing on tension-type headaches and migraines would also be valuable to better understand the unique associations and mechanisms underlying each headache subtype. Furthermore, investigating the role of confounding factors such as dietary habits, comorbid conditions, and genetic predispositions could provide deeper insights into the relationship between IDA and chronic headaches.

## 5. Conclusion

This systematic review and meta-analysis demonstrate a significant association between IDA and chronic headache disorders, with a pooled prevalence of chronic headaches in IDA patients at 38% and IDA in chronic headache patients at 20%. IDA was associated with a 76% higher risk of chronic headaches. These findings highlight the importance of routine screening for IDA, particularly in high-risk groups such as women of reproductive age. Despite robust findings, limitations include significant heterogeneity and the observational nature of included studies. Future research should focus on longitudinal studies to establish causality, evaluate the impact of iron supplementation on headache outcomes, and explore other anemia types and confounding factors to refine clinical management strategies.

## Figures and Tables

**Table 1:** Summary characteristics of studies reporting chronic headaches among patients with IDA.

Study	Country	Study design	Age (mean years)	Male %	Type of anemia	Key findings
Aamodt et al., 2004 [17]	Norway	Cross-sectional study	37.5	0	IDA	Weak association between hemoglobin levels and headaches; significant findings in certain subgroups.
Adinma and Agbai 2000 [9]	Nigeria	Prospective observational study	27.05	0	IDA	Significant association between postpartum headache and anemia, potentially linked to hormonal changes postdelivery.
Ghasami, Faraji, and Mohammadbeigi, 2012 [10]	Iran	Quasicontrol clinical trial study	29.31	0	IDA	Iron tablets significantly reduce the number of migraine attacks and painkiller usage.
Pamuk et al., 2016 [18]	Turkey	Cross-sectional study	38.8	0	IDA	Increased frequency of migraine and headaches in IDA patients, with associated anxiety. Physicians should consider anxiety and depression in IDA patients with unexplained symptoms.
Sari and Kama Başci 2024 [11]	Turkey	Retrospective cross-sectional study	43.3	14.6	IDA	Anemia and iron deficiency do not affect migraine frequency/severity, except in menstrual migraine. Ferritin levels may influence migraine treatment response.

**Table 2:** Summary characteristics of studies reporting IDA among patients with chronic headaches.

Study	Study design	Country	Age (mean years)	Male %	Type of anemia	Definition of migraine	Key findings
Eidlitz-Markus et al., 2017 [19]	Retrospective cohort study	Israel	11.6	46.4	Any IDA	International Classification of Headache Disorders (ICHD-III-beta)	Migraine in children is linked to more organic comorbidities, while tension-type headache is linked to nonorganic comorbidities. A multidisciplinary approach is recommended.
Ozmen and Ozcan, 2014 [20]	Prospective case-control study	Turkey	Not specified	Not specified	IDA	International Classification of Headache Disorders-II (ICHD-II)	IDA is prevalent in migraine patients but not in those with tension-type headaches.
Gür-Özmen and Karahan-Özcan, 2016 [12]	Prospective case-control study	Turkey	TTH = 36.5; control = 35.8; migraine = 35.05	10.2	IDA	International Classification of Headache Disorders-II (ICHD-II)	IDA is significantly associated with menstrual migraine, suggesting it as a potential comorbidity.
Lateef et al., 2009 [13]	Cross-sectional study	US	12.22	49.79	IDA	Not specified	Headaches are strongly associated with asthma, hay fever, and ear infections in children. Anemia is more common in children with headaches.
Ozcelik et al., 2023 [21]	Retrospective cohort study	Turkey	41.24	29.03	IDA	At least 1 year of migraine	IDA and other comorbidities are associated with chronic migraine. Various triggers are frequently reported in these patients.
Singh et al., 2023 [3]	Prospective case-control study	India	51.1	35.71	IDA	International Classification of Headache Disorders (ICHD-3)	IDA is independently associated with CDH severity but not with its type, subtype, or duration. Screening for IDA in CDH patients is recommended.
Tayyebi et al., 2019 [22]	Prospective case-control study	Iran	36.28	24	IDA	IHS–based migraine criteria	There is a significant relationship between IDA and migraine, particularly in women and girls. Treatment of IDA may help in managing migraines.
Vukovic-Cvetkovic et al., 2010 [23]	Prospective cohort study	Germany	39.9	0	IDA	International Classification of Headache Disorders-II (ICHD-II)	IDA is more common in women with menstrual migraines. IDA should be checked and treated in these patients.

## Data Availability

All data generated or analyzed during this study are included in this published article (and its Supporting information files).

## Supporting Information

Figure S1: Meta-regression showing heterogeneity in studies of chronic headache in patients with IDA.

Figure S2: Meta-regression showing heterogeneity in studies of IDA in patients with chronic headaches.

Figure S3: Leave-one-out analysis representing the prevalence of chronic headaches among patients with IDA.

Figure S4: Leave-one-out analysis representing the prevalence of IDA among patients with chronic headaches.

Figure S5: Baujat plot showing heterogeneity in studies of chronic headache in patients with IDA.

Figure S6: Baujat plot showing heterogeneity in studies of IDA in patients with chronic headaches.

Figure S7: Funnel plots depicting the publication bias of risk of chronic headache in patients with IDA.

Figure S8: Funnel plots depicting the publication bias of risk of IDA in patients with chronic headaches.

Table S1: PRISMA checklist.

Table S2: Inclusion and exclusion criteria.

Table S3: The adjusted search term as per the searched electronic database.

Table S4: NOS for the quality assessment of included studies.
